# The Relationship Between Plasma Tetrahydrocannabinol Levels and Intraocular Pressure in Healthy Adult Subjects

**DOI:** 10.3389/fmed.2021.736792

**Published:** 2022-01-17

**Authors:** Sameh Mosaed, Andrew K. Smith, John H. K. Liu, Donald S. Minckler, Robert L. Fitzgerald, David Grelotti, Emily Sones, Robert N. Weinreb, Thomas D. Marcotte

**Affiliations:** ^1^Gavin Herbert Eye Institute, University of California, Irvine, Irvine, CA, United States; ^2^Irvine School of Medicine, University of California, Irvine, Irvine, CA, United States; ^3^Viterbi Family Department of Ophthalmology, University of California, San Diego, San Diego, CA, United States; ^4^Department of Pathology, University of California, San Diego, San Diego, CA, United States; ^5^Department of Psychiatry, University of California, San Diego, San Diego, CA, United States

**Keywords:** tetrahydrocannabinol (THC), glaucoma, cannabis, intraocular pressure (IOP), treatment

## Abstract

**Background:**

Δ9-tetrahydrocannabinol (THC) has been shown to decreased intraocular pressure (IOP). This project aims to define the relationship between plasma THC levels and IOP in healthy adult subjects.

**Methods:**

Eleven healthy subjects received a single dose of inhaled cannabis that was self-administered in negative pressure rooms. Measurements of IOP and plasma THC levels were taken at baseline and every 30 min for 1 h and afterwards every hour for 4 h. IOP reduction and percent change in IOP over time were calculated. Linear regression models were used to measure the relationship between IOP and plasma THC levels. Two line linear regression models with F-tests were used to detect change points in the regression. Then, Pearson correlations were computed based on the change point.

**Results:**

Twenty-two eyes met inclusion criteria. The average peak percentage decrease in IOP was 16% at 60 min. Percent IOP reduction as well as total IOP reduction demonstrated a negative correlation with THC plasma levels showing r-values of −0.81 and −0.70, respectively. *F*-tests revealed a change point in the regression for plasma levels >20 ng/ml. For levels >20 ng/ml, the correlation coefficients changed significantly with *r*-values of 0.21 and 0.29 (*p* < 0.01).

**Conclusion:**

Plasma THC levels are significantly correlated with IOP reduction up to plasma levels of 20 ng/ml. Plasma levels >20 ng/ml were not correlated with further decrease in IOP. More research is needed to determine the efficacy of THC in reducing IOP for eyes with ocular hypertension and glaucoma.

## Introduction

Although not yet adopted in clinical practice, prior studies have demonstrated the intraocular pressure (IOP)-lowering effects of cannabis (1–4). Inhaled marijuana has been shown to decrease IOP by 25% in some studies while intravenous THC lowered IOP by 37% (3, 4). Topically applied THC in animal models and sublingual THC in humans have likewise demonstrated significant IOP-lowering effects (1, 2). Despite the IOP-lowering effects of cannabis, clinical adoption of THC for the treatment of elevated IOP has been limited.

There are several reasons for this, namely that topically administered pharmaceutical formulations of THC have historically poor corneal penetration, and hence limited IOP-lowering effects, and systemic administration is associated with psychotropic and potential cardiovascular side effects, thereby limiting use in clinical practice (5–9). Improving corneal penetration is an area of active research and some have shown promise through various methods (10–14).

When inhaled, THC is detected in the serum within seconds after the first puff, achieving peak plasma levels within 3–10 min (15). The bioavailability of THC varies greatly, with ranges reported between 2 and 56% (16). This variability is attributed to the differences in smoking practices and is influenced by the depth of inhalation, number of puffs, time between puffs as well as hold time of each user. After stopping inhalation, plasma levels fall rapidly. For example, when subjects smoked a cigarette containing 3.55% THC, peak concentrations ranged from 76 to 267 ng/ml but were <5 ng/ml within 2 h for all subjects (17). The average plasma clearance has been reported to be 11.8 ± 3 L/h for women and 14.9 ± 3.7 L/h for men with plasma half-life ranging from 18.7 h to 4.1 days (18, 19). The speed at which THC leaves the serum is attributed to its wide distribution into tissues including brain, heart, lungs, and adipose tissue as well as its metabolism by the liver (20). Metabolism by P450 enzymes in the liver turn the compound into a number of different metabolites including most prominently THC-COOH (17). These metabolites are then excreted in the feces and urine.

Recently, the first double-blinded randomized controlled trial demonstrated that inhaled THC reduced IOP by 16% in healthy adult subjects (21). This study reports on the correlation between THC plasma levels and IOP reduction of those patients. This is the first study to assess the correlation between THC plasma levels and IOP in adult healthy subjects.

## Materials and Methods

### Regulatory Process

This study was approved by the University of California, San Diego (UCSD) Human Research Protections, and adhered to the Declaration of Helsinki. The parent study was conducted under IND 131268, and approved by the Research Advisory Panel of California. This study was conducted at the University of California, Center for Medicinal Cannabis Research (CMCR). Two rooms were specifically outfitted with a negative pressure system to enable cannabis to be vented to the atmosphere without contaminating the workspace of others. Cannabis was harvested at the University of Mississippi under the supervision of the National Institute on Drug Abuse (NIDA).

### Participant Selection

Informed consent was obtained from all participants. Participants were a consecutive subset of individuals enrolled in a larger study examining the impact of acute cannabis inhalation on driving performance. Subjects were recruited from the community. Eligible participants (healthy adults between 21 and 55 years of age) were scheduled for a baseline session and one, 8-h experimental session at the CMCR.

Patients were included who were older than 21 years, a licensed driver and driven a minimum of 1,000 miles in the past year, a regular cannabis smoker (>/=4 times in the past month), willing to not disclose details of the simulator and iPad-based assessments, and willing to complete the IOP tonometer evaluations. Exclusion criteria for this study included a known history of glaucoma or other eye disorder other than refractive error, the inability to refrain from contact lens use on the day of visits, history of traumatic brain injury, an unwillingness to abstain from cannabis for: 2 days prior to screening visit (so driving simulation will not be impaired) and 2 days prior to experimental visit (2–3 half-lives of THC), a positive pregnancy test, a positive result on toxicity screening for cocaine, amphetamines, opiates, and phencyclidine (PCP). However, a positive result for a prescribed or recommended drug (cannabis) was not exclusionary. Individuals with current substance use disorders as assessed using the Drug Abuse Screening Test (DAST) and Alcohol Use Disorders Identification Test (AUDIT) were excluded. Subjects were also excluded for being unwilling to be transported by cab or have a responsible adult drive them home after experimental session or inability to complete study procedures (i.e., poor veins, unwillingness to be transported home by taxi, or friend).

### Study Design

Three-hundred participants were recruited with the intention to study up to 220 participants who met inclusion/exclusion criteria and ultimately provide complete data. Eleven participants were screened and enrolled in the IOP component. At the beginning of the screening/baseline visit and the experimental visit, subjects underwent a urine drug screen and breathalyzer for alcohol and drugs. In addition, an oral fluid sample was run for the presence of delta-9 THC using a testing device (Draeger 5000) which identifies THC levels at above vs. below 5 ng. A positive reading on the Draeger was considered indicative of use within the past day. Any participants with a positive reading were excluded (none occurred within the subsample for this study).

Participants were divided into two groups and each received either a 5.9 or 13.4 w/w % cannabis cigarette at their visits. Group assignment were assigned using a permuted blocks randomization with stratification by prior cannabis exposure [frequent user (>4x per week) vs. occasional user (<4x per week)]. They were asked to smoke 700 mg cigarettes with either 5.9, or 13.4% (at the beginning of the day, and to measure IOP, complete driving simulations, iPad-based performance assessments, and bodily fluid draws [e.g., blood, oral fluid (OF) saliva, breath] before the cannabis smoking and over the subsequent 6 h after cannabis smoking. Participants were instructed to “*smoke the joint/cigarette the way you do at home to get high”* (i.e., there was no requirement that they finish the entire cigarette). Though not mandatory to incinerate the cigarette to the proximal tip, a minimum of 4 puffs was required for a participant to remain in the study. They were allowed 10 min to smoke. The allocation schedule was kept in the pharmacy and concealed from other study personnel. Patients and assessors were blinded to group assignments.

### Monitoring of Vital Signs

Vital signs were monitored throughout the experiment at hourly intervals to monitor the subject's health status as well as to quantify marijuana's general effects. At any sign of an adverse reaction (e.g., a change in blood pressure or pulse rate or development of psychological distress), an investigator was called. Subjects remained in the laboratory under direct observation for 7 h after the marijuana smoking inhalations were completed. At that time, a final vital sign and self-report status check was made and upon satisfactory readings, the subject was released and driven back to his/her domicile by taxicab or prearranged transportation. The return transport procedure was observed directly by staff to ensure compliance.

### Cardiovascular Monitoring

Blood pressure and pulse were assessed pre-smoking, and at approximately every 30 min for 2 h post-smoking session, then up to every hour for the additional 3 h.

### IOP and THC Plasma Monitoring

THC plasma levels were taken prior to smoking, and then at ~12 min after, 40, 80, 120 min, and every hour for the additional 3 h after smoking. The average of three IOP readings at each of these time points were taken for each eye. IOP readings were obtained by trained research assistants. Measurements of IOP were taken using the non-contact Ocular Response Analyzer (Reichert Technologies, Depew, NY). This device is FDA approved and was used in our protocol in accordance to the FDA label. Of note, if the participant had high IOP (21 mmHg or higher) prior to smoking, we recommended follow-up with an ophthalmologist.

### Data Analysis Overview

In this analysis, data from the low-dose and high-dose group were combined as there were no statistically significant differences in the plasma levels between the two groups. This is because the participants were permitted to self-administer the quantity of puffs until they felt a psychotropic effect. Total IOP reduction in mmHg was calculated along with the percent reduction for each participant. The peak THC plasma level was determined for each participant. Linear regression models were then used to assess the relationship between IOP and THC plasma levels. Two-line linear regression models with *F*-tests were used to detect change points in the regression models. Pearson correlations were then calculated for values under and over the change point.

## Results

**Figure 1:**
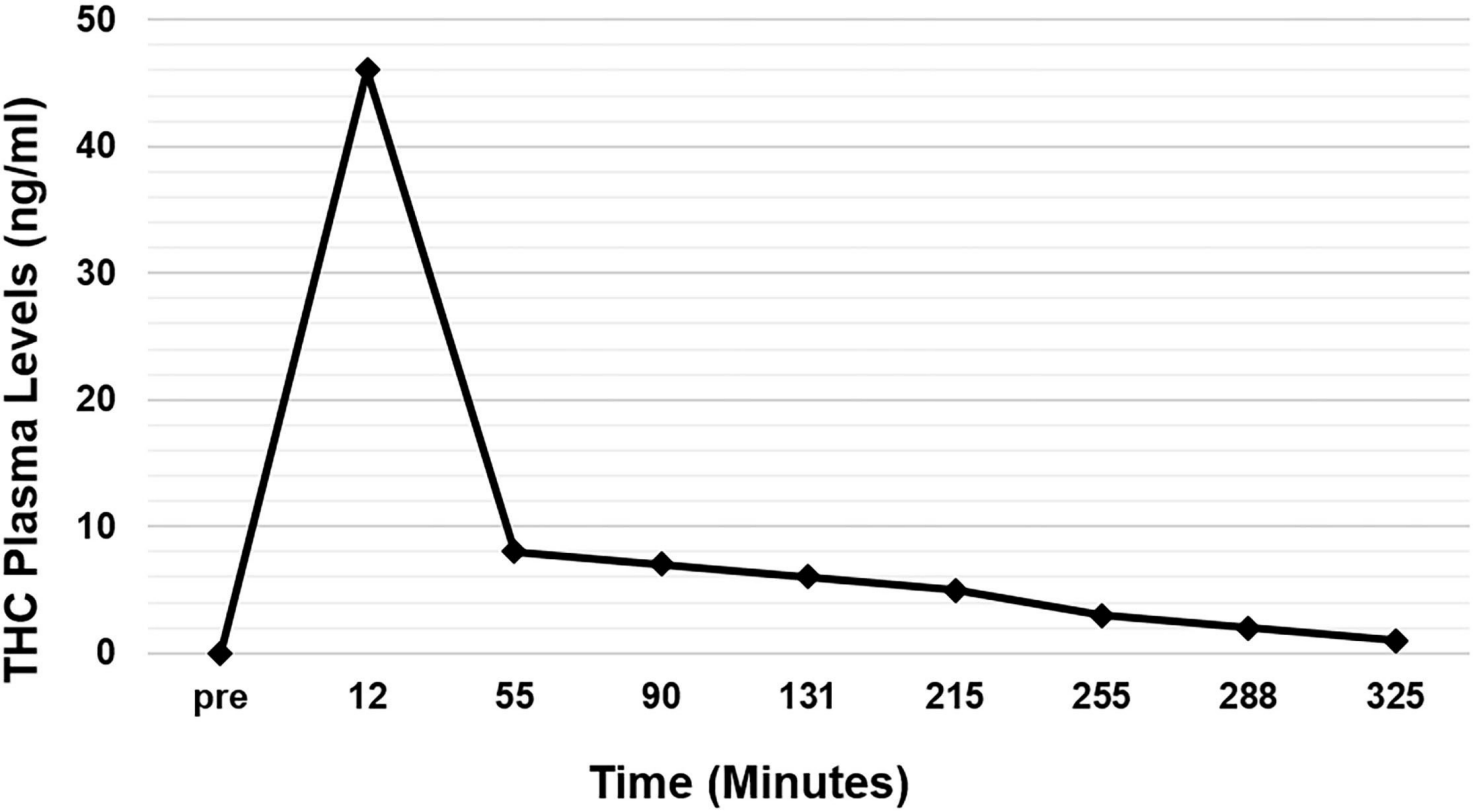
Average THC plasma levels over time.

**Figure 2:**
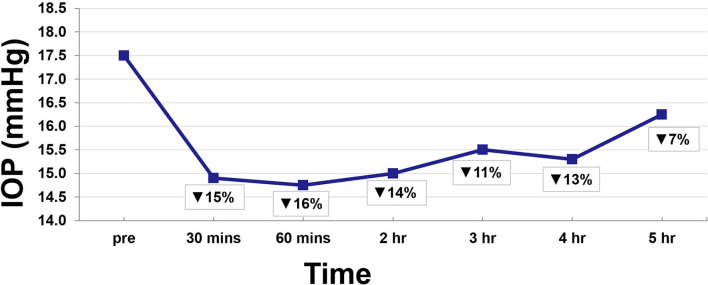
Average IOP reduction over time.

Twenty-two eyes of 11 subjects were included in the analysis. There are no missing values or outliers in the data. The IOP was normally distributed. The average peak THC plasma level was 45 ng/ml and occurred at 12 min. THC plasma levels spiked reaching peak levels at 12 min and then rapidly declined, achieving levels of <10 ng/ml by 55 min (Figure 1). THC levels continued to gradually decline for the remaining time periods. Average IOP before inhalation was 17.5 mmHg. After inhalation, IOP percent reduction ranged from 7 to 16% with the greatest IOP percent reduction of 16% seen at 60 min (Figure 2). This percent reduction of IOP gradually decreased for the remaining time periods.

**Figure 3:**
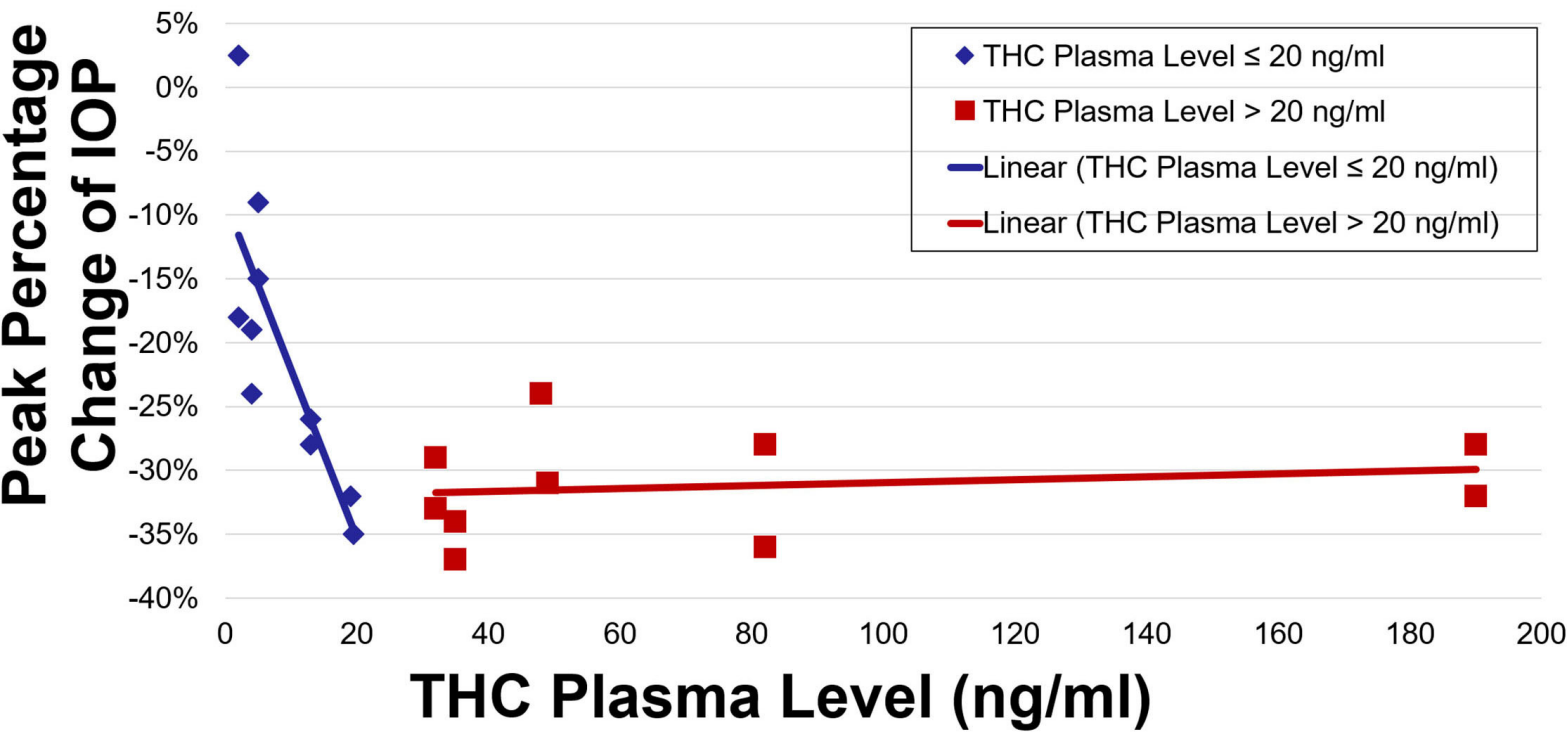
Peak percentage change of IOP by THC plasma level.

**Figure 4:**
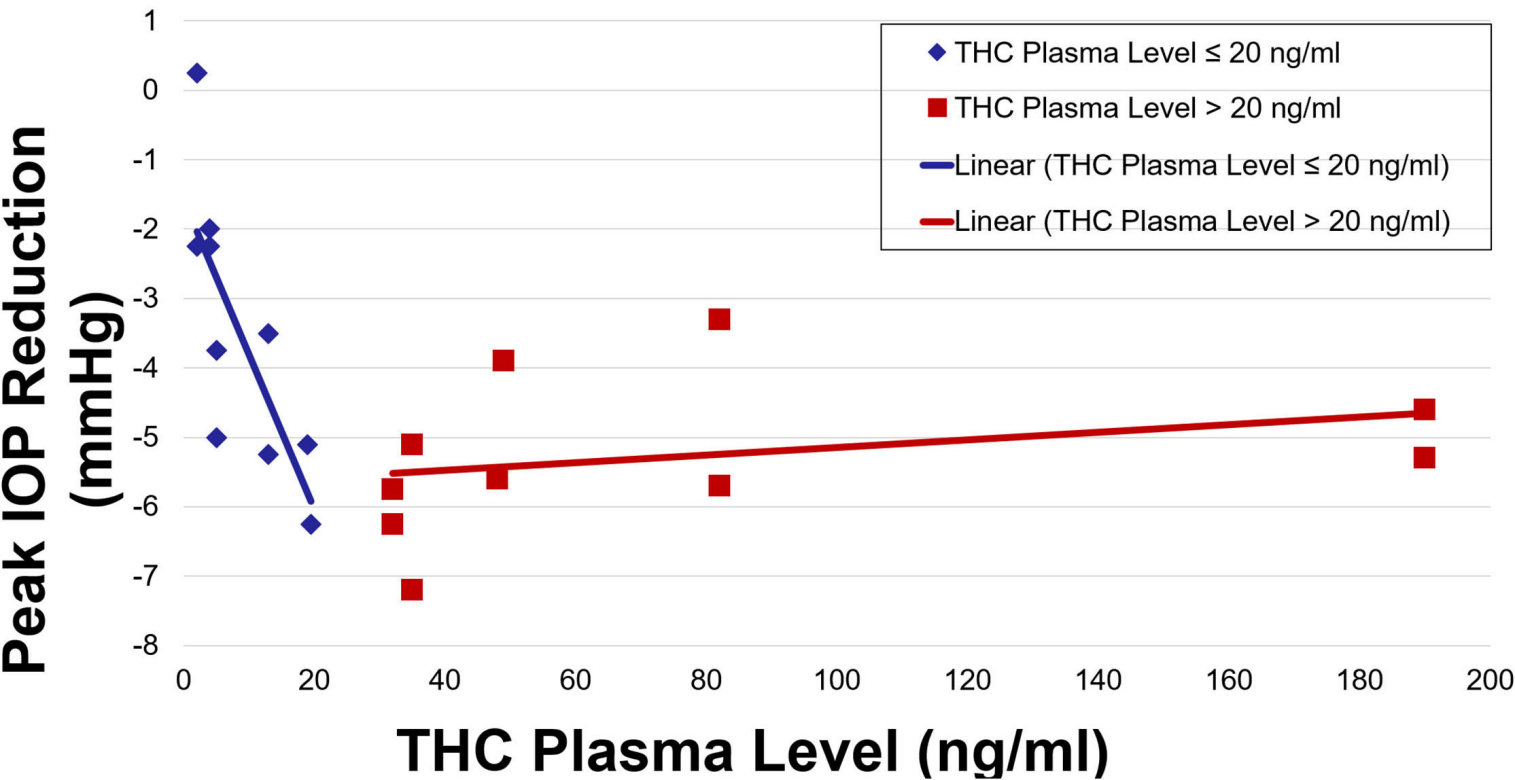
Peak IOP reduction by THC plasma level.

IOP and plasma THC levels showed a strong negative correlation (Figures 3, 4). Two line linear regression models revealed a change point at 20 ng/ml. Percent IOP reduction and THC plasma levels demonstrated a correlation coefficient of −0.81 for THC plasma levels up to 20 ng/ml and 0.21 for levels over 20 ng/ml (*F*-statistic = 16.93, *p* < 0.01). Total IOP reduction and plasma THC showed similar results with correlation coefficients of −0.70 for THC plasma levels up to 20 ng/ml and 0.29 for values over 20 ng/ml (*F*-statistic = 7.92, *p* < 0.01).

## Discussion

The results of this study indicate that in healthy adult subjects, inhaled THC significantly lowers IOP, and that this effect correlates with plasma THC levels. IOP reduction occurred soon after inhalation and was reduced by as much as 16%. Both percent IOP reduction and absolute IOP reduction in mmHg demonstrated a strong negative correlation with plasma THC levels. The IOP was lowered significantly for 4 h after inhalation. Furthermore, increasing plasma levels up to a concentration of 20 ng/ml was strongly correlated with increasing reduction in IOP. THC plasma levels >20 ng/ml were not correlated with further reduction in IOP.

Consistent with prior descriptions of THC metabolism, participants demonstrated a rapid spike in plasma THC levels that quickly decreased (15, 20). At 30 min, IOP reduction was already at 15%, but this reduction continued ranging from 11 to 16% for 4 h despite the fall in THC plasma levels (Figure 2). Indeed, THC plasma levels are known to decrease quickly as the lipophilic substance leaves the serum and deposits in the tissues of the body where it exerts its various effects (16, 17). As such, IOP reduction continued despite the rapid fall in serum levels.

Correlation between THC plasma levels and its other effects in the body have been described. The lipophilicity of THC results in a rapid withdrawal from the serum into the tissues, causing a situation where THC effects correlate with early THC plasma levels rather than concurrent THC plasma levels (22). For example, psychotropic effects have been correlated with THC plasma levels during the first 4 h after inhalation (23). Furthermore, models for predicting effects on heart rate, alertness, and psychotropic effects have been developed to predict the degree of these effects based on THC plasma levels (22). They suggest plasma levels above which additional effect on the body are less likely or impactful. This study shows similar results for IOP, and represents the first analysis to describe IOP reduction as it correlates with THC plasma levels. It further suggests a plasma level of 20 ng/ml as a target plasma level above which additional IOP-lowering is not strongly correlated.

The specific mechanisms by which cannabis lowers IOP are the subject of active investigation. It is known that there are cannabinoid receptors located throughout the eye, in particular in the ciliary muscle, ciliary epithelium, trabecular meshwork, and Schlemm's canal (24). These receptors, part of the endocannabinoid system, result in a series of varied changes such as ciliary body contraction, widening of Schlemm's canal, and activation of matrix metalloproteinase, which enhances outflow of the trabecular meshwork (25, 26). Moreover, cannabinoids also upregulate COX-2, potentially increasing the presence of prostaglandin E2 and metalloproteinases, enhancing the outflow of aqueous humor, and reducing IOP (27, 28). It has also been suggested that some cannabinoids lower IOP through adrenergic receptors within the eye as well as through a mechanism involving prostaglandins by action of endocannabinoid metabolites (29–31). Besides any manipulation on ocular blood flow, the potential role of cannabis in neuroprotection has been suggested by many studies, although no clear evidence of neuroprotection in glaucoma has yet been established (32, 33).

Given the wide range of systemic considerations, the routine use of inhaled or ingested cannabis for glaucoma treatment has not been clinically practical. However, novel compounds with improved corneal penetration are being developed for topical administration, thereby mitigating systemic side-effects (10–14, 34). Non-psychotropic cannabinoids and other CB1 receptor targets are being investigated for potential treatments that avoid systemic effects (35, 36). In addition, formulations are being developed to improve duration of action (37).

This study is not without limitations. First, the method of obtaining the IOP data was through a non-contact tonometry method, thereby facilitating the acquisition of IOP data in contact lens wearers, and decreasing the invasiveness of measurements for this pilot study. Future studies would ideally use more consistently accurate methods of tonometry. In addition, this study only involved healthy normal adults, and does not characterize the IOP-lowering effects of marijuana in subjects with glaucoma, ocular hypertension, or in subjects with concomitant IOP-lowering medications. Future studies can also focus on IOP-lowering response based on patient characteristics such as sex, ethnicity, etc…

In conclusion, the current study demonstrates a strong correlation between IOP and THC plasma levels. This study further suggests that a peak THC plasma level above 20 ng/mL is not correlated with further IOP reduction, and that non-physiologic IOP levels are not seen with increasing plasma levels of THC in healthy subjects. Defining the role of cannabis in glaucoma treatment requires further studies to better characterize these effects in different patient populations.

## Data Availability Statement

The raw data supporting the conclusions of this article will be made available by the authors, without undue reservation.

## Ethics Statement

The studies involving human participants were reviewed and approved by University of California, San Diego (UCSD) Human Research Protections. The patients/participants provided their written informed consent to participate in this study.

## Author Contributions

SM, JL, RF, TM, RW, and DM contributed to the conception and design of the study. AS, DG, and ES organized and analyzed the data as well as performed the statistical analysis. AS wrote the first draft of the manuscript. SM, ES, and DG wrote the methods section of the manuscript. All authors contributed to the revising and editing of the manuscript and approved the submitted version of the manuscript.

## Funding

This study was conducted using departmental funds from the University of California, Irvine, Gavin Herbert Eye Institute.

## Conflict of Interest

The authors declare that the research was conducted in the absence of any commercial or financial relationships that could be construed as a potential conflict of interest.

## Publisher's Note

All claims expressed in this article are solely those of the authors and do not necessarily represent those of their affiliated organizations, or those of the publisher, the editors and the reviewers. Any product that may be evaluated in this article, or claim that may be made by its manufacturer, is not guaranteed or endorsed by the publisher.
